# Early detection of metachronous bile duct cancer in Lynch syndrome: report of a case

**DOI:** 10.1007/s00595-013-0669-3

**Published:** 2013-07-31

**Authors:** Kunitoshi Shigeyasu, Kohji Tanakaya, Takeshi Nagasaka, Hideki Aoki, Toshiyoshi Fujiwara, Kokichi Sugano, Hideki Ishikawa, Teruhiko Yoshida, Yoshihiro Moriya, Yoichi Furukawa, Ajay Goel, Hitoshi Takeuchi

**Affiliations:** 1 Department of Surgery, Iwakuni Clinical Center, Yamaguchi, Japan; 2 Department of Gastroenterological Surgery, Okayama University Graduate School of Medicine, Dentistry and Pharmaceutical Sciences, 2-5-1 Shikata-cho, Kita-ku, Okayama, Okayama 700-8558 Japan; 3 Oncogene Research Unit Cancer Prevention Unit, Tochigi Cancer Center Research Institute, Tochigi, Japan; 4 HNPCC Registry and Genetic Testing Project of the Japanese Society for Cancer of the Colon and Rectum (JSCCR), Tokyo, Japan; 5 Department of Molecular-Targeting Cancer Prevention, Kyoto Prefectural University of Medicine, Osaka, Japan; 6 Genetics Division, National Cancer Center Research Institute, Tokyo, Japan; 7 Department of Surgery, National Cancer Center Hospital, Tokyo, Japan; 8 Division of Clinical Genome Research, Advanced Clinical Research Center, Institute of Medical Science, The University of Tokyo, Tokyo, Japan; 9 Division of Gastroenterology, Department of Internal Medicine, Charles A Sammons Cancer Center and Baylor Research Institute, Baylor University Medical Center, Dallas, TX USA

**Keywords:** Lynch syndrome, Bile duct cancer, Surveillance, Early detection

## Abstract

Lynch syndrome is an autosomal dominant disease associated with a high incidence of colorectal, endometrial, stomach, ovarian, pancreatic, ureter and renal pelvis, bile duct and brain tumors. The syndrome can also include sebaceous gland adenomas and keratoacanthomas, and carcinoma of the small bowel. The lifetime risk for bile duct cancer in patients with Lynch syndrome is approximately 2 %. The present report describes a case of Lynch syndrome with metachronous bile duct cancer diagnosed at an early stage. The patient was a 73-year-old Japanese male who underwent a successful left lobectomy of the liver, and there was no sign of recurrence for 2 years postoperative. However, this patient harbored a germline mutation in *MLH1*, which prompted diagnostic examinations for noncolorectal tumors when a periodic surveillance blood examination showed abnormal values of hepatobiliary enzymes. Although most patients with bile duct cancer are diagnosed at an advanced stage, the bile duct cancer was diagnosed at an early stage in the present patient due to the observation of the gene mutation and the preceding liver tumor. This case illustrates the importance of continuous surveillance for extracolonic tumors, including bile duct cancer, in patients with Lynch syndrome.

## Introduction

Lynch syndrome, also known as hereditary nonpolyposis colorectal cancer (HNPCC), is an inherited autosomal dominant disorder associated with germline mutations in genes involved in DNA mismatch repair (MMR), including *MLH1*,* MSH2*, *MSH6* and *PMS2* [1]. Although variable, the lifetime risk of developing colorectal cancer (CRC) in patients with Lynch syndrome is approximately 80 %, with male carriers having a higher cumulative risk than female carriers [2]. Carriers of these mutations also have an increased lifetime risk of developing extracolonic tumors, such as endometrial, stomach, ovarian, pancreas, ureter and renal pelvis, bile duct and brain (usually glioblastoma) tumors. Further, the syndrome may also include sebaceous gland adenomas, keratoacanthomas, and carcinoma of the small bowel, although these occur at a somewhat lower incidence than the other neoplasms [3].

Since the 1980s, conventional surveillance by colonoscopy has been recommended for Lynch syndrome families. Some studies have shown that periodic examination by colonoscopy can detect CRC at an early stage, leading to a 62 % reduction in the risk of malignancy and a significant reduction in mortality associated with CRC [4–8]. While screening may be effective for the early detection of endometrial cancer in these patients [9–11], the efficacy of surveillance for other forms of co-occurring tumors has not yet been established.

The present report describes the case of a patient with Lynch syndrome in whom bile duct cancer was detected in its early stages after abnormal hepatobiliary function was detected during routine surveillance, which prompted additional diagnostic measures.

## Case presentation

**Fig. 1:**
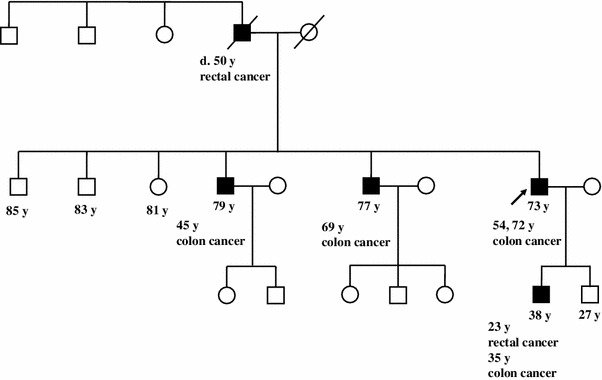
The patient’s pedigree. The patient’s family satisfied the revised Amsterdam criteria for the diagnosis of Lynch syndrome

A 73-year-old male with a history of synchronous and metachronous CRCs had undergone right hemicolectomy with partial liver resection at 54 years of age for synchronous ascending and transverse colonic tumors (three lesions) with a single liver metastasis (Stage IV: Union Internationale Contre le Cancer, UICC TNM staging). At 72 years, he developed metachronous sigmoid colon cancer (Stage I), which was treated by surgical resection. Although the age of CRC onset was relatively late, his family history met the revised Amsterdam criteria for the diagnosis of Lynch syndrome (Fig. 1); his father developed rectal cancer at 50 years of age, his elder brother had developed advanced CRC when he was 45, another brother developed CRC when he was 69 and one of his sons had developed metachronous CRCs at 23 and 35 years of age. After obtaining informed consent, genetic testing for MMR identified a germline mutation in *MLH1*, c.209_211delAAG. Because this deletion is localized near the 5′ site of exon 3, a reverse transcriptase polymerase chain reaction (RT-PCR) analysis was performed and revealed exon 3 (279 bp) skipping. Thereafter, the patient was enrolled in a surveillance program for Lynch syndrome (colonoscopy, gastroduodenoscopy, abdominal ultrasound, urinalysis and urine cytology every year) with a routine postsurgical examination protocol (periodic abdominal ultrasonography and computed tomography every 6 months and blood examinations every 3 months).

**Fig. 2:**
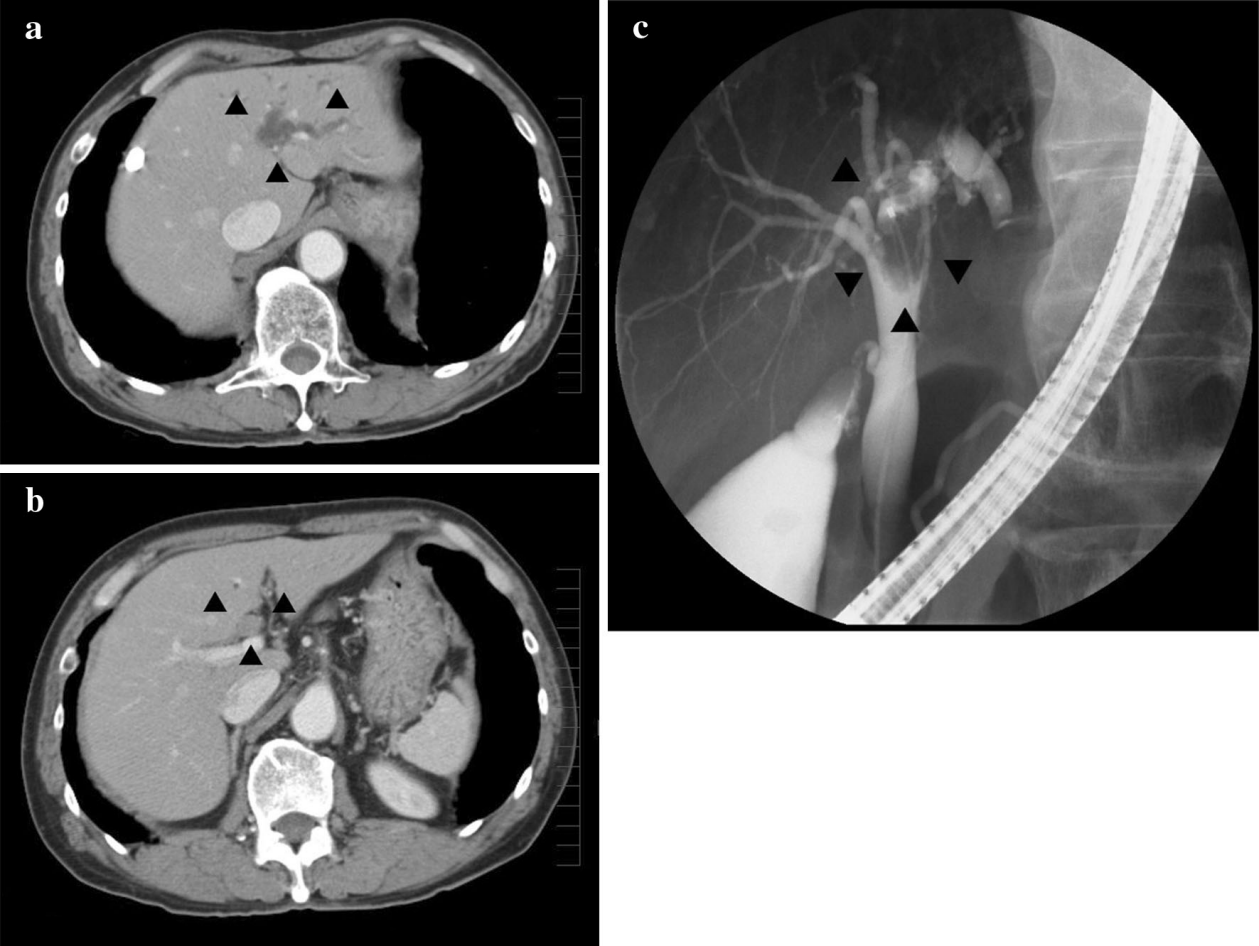
Imaging of the hepatobiliary system. Computed tomography (**a**, **b**) and endoscopic retrograde cholangiography (**c**) revealed a mass in the left hepatic bile duct and a dilated left intrahepatic bile duct (*arrowheads*)

One year after the surgical resection for Stage I sigmoid colon cancer, a periodic examination revealed increased values of hepatobiliary enzymes in his peripheral blood sample, with aspartate aminotransferase of 53 U/L (normal range 13–33 U/L), alanine aminotransferase of 109 U/L (normal range 6–27 U/L) and alkaline phosphatase of 635 U/L (normal range 115–359 U/L). His total bilirubin level was also slightly elevated at 1.6 mg/dL (normal range 0.3–1.28 mg/dL) (Table 1). Further examinations were performed to screen for Lynch syndrome-related tumors. Abdominal ultrasonography revealed dilatation of the left intrahepatic bile duct. Computed tomography and endoscopic retrograde cholangiography revealed a tumor in the left hepatic bile duct and dilatation of the left intrahepatic bile duct, respectively (Fig. 2). He underwent surgical resection for a presumed diagnosis of bile duct cancer.

**Table 1:** Laboratory data

Parameters	Normal range	Units	3 months before operation	Just before operation	3 months after operation
Total bilirubin	0.3–1.28	mg/dl	1.2	1.6	1.2
Aspartate aminotransferase	13–33	U/L	31	53	19
Alanine aminotransferase	6–27	U/L	48	109	16
Alkaline phosphatase	115–359	U/L	ND	635	347

**Fig. 3:**
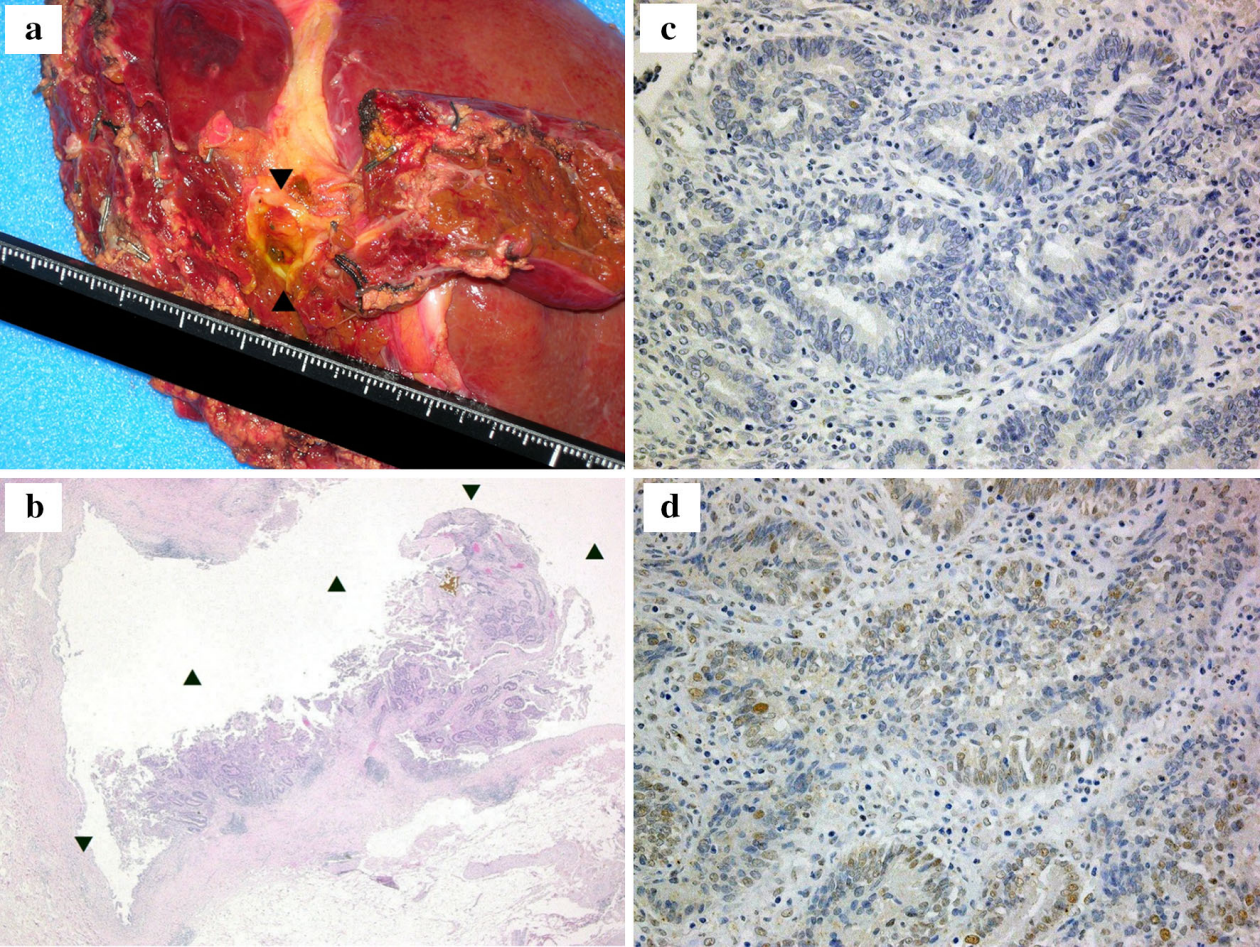
The results of a histopathological analysis of the tumor sample obtained from the left lobectomy. **a** The gross appearance of the resected tissue. **b** The H&E staining (×100) revealed a well-differentiated papillary hilar bile duct cancer [T1 (fibromuscular layer, fm) N0M0 Stage IA (Union Internationale Contre le Cancer, UICC TNM staging) (*arrowheads*)]. **c** Immunostaining of the tumor for MLH1 (×400). **d** Immunostaining of the tumor for MSH2 (×400). Immunoreactivity was observed for MSH2, but not for MLH1

**Fig. 4:**
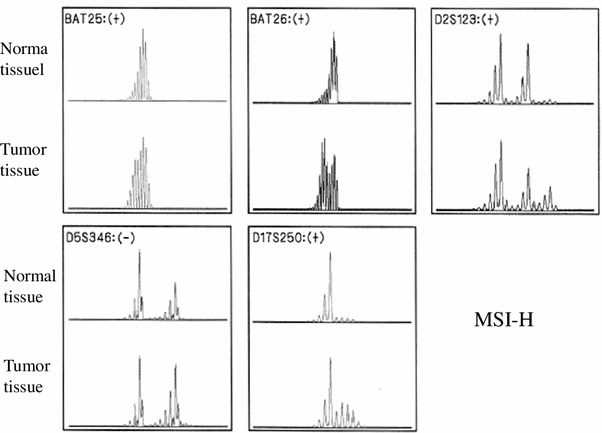
The results of a microsatellite instability (MSI) analysis. The microsatellite instability (MSI) analysis showed that the tumor was MSI high according to the National Cancer Institute panel (NCI panel: D2S123, D5S346, D17S250, BAT25, and BAT26); replication errors were observed in four microsatellite markers [MSI-H (4/5)]. +, −: Existence of replication errors in each microsatellite marker

Intraoperative cholangioscopy detected a papillary tumor at the origin of the left hepatic duct. A histological examination confirmed a well-differentiated papillary adenocarcinoma of the bile duct [T1 (fibromuscular layer, fm) N0M0 Stage IA (UICC TNM staging)]. As expected, immunohistochemical staining demonstrated that the tumor was MLH1 negative and MSH2 positive (Fig. 3), which was in agreement with the germline mutation in *MLH1*. A microsatellite instability (MSI) analysis diagnosed the tumor as MSI high using the National Cancer Institute panel (NCI panel: D2S123, D5S346, D17S250, BAT25 and BAT26); replication errors were observed in four microsatellite markers [MSI-H (4/5)] (Fig. 4).

## Discussion

The Bethesda guidelines, proposed in 1996 and revised in 2002, are probably the most important clinical guidelines for Lynch syndrome, and include Lynch syndrome-associated tumors, such as colorectal, endometrial, stomach, ovarian, pancreas, ureter and renal pelvis, bile duct and brain tumors, as well as sebaceous gland adenomas, keratoacanthomas and carcinoma of the small bowel [3]. The lifetime risk for biliary tract cancer in patients with Lynch syndrome is approximately 2 % [2]. Mecklin et al. [12] described 11 cases of bile duct cancer in Lynch syndrome families; the mean age at diagnosis was 56.9 years. Of these 11 cases, seven tumors arose in the common bile duct, whereas four originated in the intrahepatic bile duct.

**Fig. 5:**
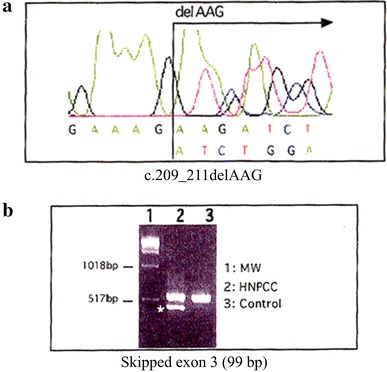
The results of the genetic analysis of *MLH1*. **a** Direct sequencing of *MLH1* revealed that the patient harbored a germline mutation in *MLH1*, c.209_211delAAG, which resulted in exon 3 skipping. **b** Reverse transcriptase polymerase chain reaction (RT-PCR) of *MLH1* messenger RNA (mRNA) revealed a shorter product [skipping of exon 3 (99 bp)] in the present case (*lane 2* HNPCC). *MW* denotes the molecular weight marker. *Control* denotes the RT-PCR product obtained from normal *MLH1* mRNA

The International Society for Gastrointestinal Hereditary Tumors database (InSiGHT/LOVD database; http://www.insight-group.org/mutations/) contains two cases of bile duct cancer with a germline mutation in *MLH1* (deletion in exon 16, c.1732-?_1896+?del). In our case, the patient harbored a germline mutation in *MLH1* c.209_211delAAG, which resulted in exon 3 skipping (Fig. 5). Of note, this mutation has not been reported previously, although one report showed that a similar deletion of *MLH1*, c.210_213delAGAA, also leads to exon 3 skipping [13].

CRC is the most common Lynch syndrome-related tumor, with a lifetime risk of approximately 80 %. Early-stage CRC is detectable by surveillance of the colorectum, and early treatment can decrease mortality. Endometrial cancer is the next most common Lynch syndrome-related cancer, with a lifetime risk of 20–60 %, and conventional screening is useful for the early detection of this cancer [2]. Thus, established surveillance protocols are effective tools for the early detection of CRC and endometrial cancer [4–11]. In contrast, the lifetime risk of bile duct cancer in patients with Lynch syndrome is only 2 % [2]. Surveillance for bile duct cancer is not recommended for Lynch syndrome families, because there are no established strategies for the early detection of bile duct cancer. The routine surveillance protocol for Lynch syndrome in our hospital is almost the same as that recommended by the Lynch syndrome surveillance program (i.e., colonoscopy, gynecological examination, transvaginal ultrasound, aspiration biopsy, gastroduodenoscopy, abdominal ultrasound, urinalysis and urine cytology every year) reported by Vasen et al. [2]. These examinations are not specifically targeted toward early detection of bile duct cancer. However, in the surveillance of patients with Lynch syndrome and their relatives, it is important to consider the risks of developing bile duct cancer, which is higher in these patients than in the general population. Carriers of MMR gene mutations are at an elevated risk of bile duct cancer compared with the general population (standardized incidence ratio 5.94) [14, 15]. Most cases of bile duct cancers, which can rapidly progress to an advanced stage because of the lack of muscularis mucosae in the wall of the bile duct, are typically diagnosed at advanced stages and are therefore associated with much higher mortality rates than cancers that occur outside the hepatobiliary system [16].

The early detection of bile duct cancer is challenging. The most promising, noninvasive and cost-effective tools include blood examinations and abdominal ultrasonography. Blood examinations can reveal increased values of hepatobiliary enzymes in 75 % of bile duct cancer patients without jaundice [17] [18], and abdominal ultrasonography can improve the early detection rates of bile duct cancer [19]. Although blood examinations and abdominal ultrasonography are useful for detecting abdominal disease, including bile duct cancer, these modalities are not utilized frequently in the surveillance program for patients with Lynch syndrome.

In the present case, a routine postsurgical examination protocol (i.e., periodic abdominal ultrasonography and computed tomography every 6 months and blood examination every 3 months) was conducted. We consider the bile duct cancer as a Lynch syndrome-related tumor. Therefore, we immediately began diagnostic investigations for bile duct cancer when the surveillance blood examination results showed abnormal hepatobiliary function. In contrast, we would not have been able to detect the bile duct cancer at an early stage in this patient had we relied on only a traditional surveillance program for Lynch syndrome instead of our routine postsurgical examination protocol, because frequent blood examinations and abdominal ultrasonography are not included as part of the surveillance protocol for Lynch syndrome.

The present case suggests that the combination of periodic blood examinations and abdominal ultrasonography every 3–6 months may be useful for the early detection of bile duct cancer in patients with Lynch syndrome. In the present case, the patient received a successful early diagnosis and treatment of rare bile duct cancer as a result of a blood examination in the postsurgical state. Although blood examinations are not part of a routine postsurgical examination protocol for Lynch syndrome patients, as this case shows, they may be effective, and should be considered for inclusion in the surveillance for Lynch syndrome. In addition, abdominal ultrasonography is noninvasive and can visualize dilated bile ducts that are often present in patients with early bile duct cancer. Further research is needed to draw definitive conclusions regarding the utility of periodic blood examinations and abdominal ultrasonography in this context, but our present case indicates that such examinations are warranted.

## Conclusion

The present report described the case of a patient with Lynch syndrome in whom bile duct cancer was detected in its early stages after a periodic blood examination showed abnormal liver function. The assessment of the hepatobiliary enzymes and subsequent ultrasonography and computed tomography facilitated early detection of this cancer. Although most cases of bile duct cancer are diagnosed at advanced stages, the fact that this patient had a known germline mutation in *MLH1* resulted in a higher level of suspicion regarding noncolorectal tumors, which prompted the use of additional diagnostic investigations for such tumors when his ordinary blood examination suggested abnormal hepatobiliary function. This case illustrates the importance of surveillance for noncolorectal tumors in patients with Lynch syndrome. Physicians should be aware that patients with Lynch syndrome are at a higher risk of developing rare extracolonic cancers compared with the general population. Since these cancers are associated with a poor prognosis, early detection through surveillance may improve the outcomes in these patients. We conclude that the combination of periodic blood examinations and abdominal ultrasonography every 3–6 months may be useful for the early detection of bile duct cancer in Lynch syndrome patients.
